# Anti-Diabetic Effects of CTB-APSL Fusion Protein in Type 2 Diabetic Mice

**DOI:** 10.3390/md12031512

**Published:** 2014-03-13

**Authors:** Yunlong Liu, Zhangzhao Gao, Qingtuo Guo, Tao Wang, Conger Lu, Ying Chen, Qing Sheng, Jian Chen, Zuoming Nie, Yaozhou Zhang, Wutong Wu, Zhengbing Lv, Jianhong Shu

**Affiliations:** College of Life Sciences, Zhejiang Sci-Tech University, Hangzhou 310018, China; E-Mails: liuyunlong5566@163.com (Y.L.); lion8907@163.com (Z.G.); guoqingtuo@163.com (Q.G.); wangtao900121@163.com (T.W.); luconger0302@163.com (C.L.); carolynchency@163.com (Y.C.); csheng@zstu.edu.cn (Q.S.); chj1999@126.com (J.C.); wuxinzm@zstu.edu.cn (Z.N.); yaozhou@zstu.edu.cn (Y.Z.); wuwutong@gmail.com (W.W.); zhengbingl@zstu.edu.cn (Z.L.)

**Keywords:** active peptide from shark liver, cholera toxin B subunit, *Bombyx mori* pupae, type 2 diabetes mellitus, oral administration

## Abstract

To determine whether cholera toxin B subunit and active peptide from shark liver (CTB-APSL) fusion protein plays a role in treatment of type 2 diabetic mice, the CTB-APSL gene was cloned and expressed in silkworm (*Bombyx mori*) baculovirus expression vector system (BEVS), then the fusion protein was orally administrated at a dose of 100 mg/kg for five weeks in diabetic mice. The results demonstrated that the oral administration of CTB-APSL fusion protein can effectively reduce the levels of both fasting blood glucose (FBG) and glycosylated hemoglobin (GHb), promote insulin secretion and improve insulin resistance, significantly improve lipid metabolism, reduce triglycerides (TG), total cholesterol (TC) and low density lipoprotein (LDL) levels and increase high density lipoprotein (HDL) levels, as well as effectively improve the inflammatory response of type 2 diabetic mice through the reduction of the levels of inflammatory cytokines tumor necrosis factor-α (TNF-α) and interleukin-6 (IL-6). Histopathology shows that the fusion protein can significantly repair damaged pancreatic tissue in type 2 diabetic mice, significantly improve hepatic steatosis and hepatic cell cloudy swelling, reduce the content of lipid droplets in type 2 diabetic mice, effectively inhibit renal interstitial inflammatory cells invasion and improve renal tubular epithelial cell nucleus pyknosis, thus providing an experimental basis for the development of a new type of oral therapy for type 2 diabetes.

## 1. Introduction

Diabetes mellitus is a common chronic metabolic disease, usually caused by the interaction of genetic and environmental factors [1]. It is characterized by a lack of insulin secretion (relative and absolute) and insulin resistance [2], always leading to metabolism disorders of fat, protein and carbohydrate [3], and is likely to produce serious complications involving some of the vital organs, including the heart, blood vessels, nerves, eyes and kidneys as well as causing tissue lesions [4]. There are approximately 366 million people worldwide who suffer from diabetes and another 280 million people with pre-diabetes as evidenced by impaired glucose tolerance. In 2011, 4.6 million people died from diabetes, meaning one diabetes-related death every seven seconds [5]. Because of its complex disease process, there is still no known cure for diabetes, and patients need to receive lifelong treatment. Therefore, the research and development of low toxicity and long-acting diabetes drugs, has a very significant impact on the prevention and treatment of diabetes and on improving people’s quality of life. Diabetes is mainly divided into type 1 diabetes and type 2 diabetes, wherein more than 90% of all people with diabetes have type 2 diabetes. The treatment of type 1 diabetes is mainly dependent on exogenous insulin [6], whereas the treatment of type 2 diabetes often includes biguanides, sulfonylureas, α-glucosidase inhibitors, and other drugs [7]. However, traditional anti-diabetic drugs have limited efficacy, have side effects, and cannot fundamentally repair damaged islet β-cells, ultimately resulting in insulin-dependency [8].

Cholera toxin (CT) is a type of enterotoxin produced by *Vibrio cholerae* with a molecular weight of 84 kDa. It is made up of an A subunit and five identical B subunits, where A is the toxic subunit. Five identical polypeptide chains of CTB form a cyclic pentamer structure with non-covalent bonds, and the structure can specifically bind to the ganglioside (GM1) on the surface of nucleated cells, inducing membrane configuration changes, that allow the connected small molecules to move into the cell [9]. CTB molecules are the CT domain with no toxin activity, so it is an ideal oral delivery carrier [10]. Arakawa *et al.* made a proinsulin gene linked to the carboxy terminus of the CTB gene and transferred this gene to a potato which then successfully expressed the fusion protein. After the potato was fed to diabetic mice, islet inflammation was improved [11]. Limaye *et al.* created a CTB and GFP fusion protein that could be expressed in tobacco chloroplasts and fed this tobacco to the mice. The results show that the fusion protein with a green fluorescent protein tag was able to be absorbed by the intestinal epithelial cells. The special fusion protein pentamer structure enables its binding to the GM1 receptor on the cell surface, entry into phagosomes by endocytosis and then entry into the endoplasmic reticulum. GFP molecules are discharged extracellular through the Golgi into the lymphatic circulation and then further into the blood circulation [12]. 

Hepatic Stimulator Substance (HSS) is a kind of liver-specific stimulating factor originally found in the liver of weanling rats [13], has the functions of promoting liver regeneration, stimulating liver cells to synthesize DNA and mitosis [14,15], starts hepatocyte proliferation and repairs liver damage [16,17]. It acts in an organ-specific and not species-specific manner. APSL is a hepatocyte stimulating cytokine which is isolated and purified from the liver of *Chiloscyllium plagiosum*, and our previous studies demonstrated that APSL was able to significantly protect mouse islets from lesions and to reduce the FBG level in type 2 diabetic mice. In addition, APSL could protect against acute hepatic injury induced by acetaminophen or CCl_4_ [18,19,20].

In the current study, we evaluated the anti-diabetes effects of the cholera toxin B subunit and active peptide from shark liver (CTB-APSL) fusion protein in type 2 diabetic mice. We found that the CTB-APSL fusion protein has good activity against type 2 diabetes and effectively improves its complications. Therefore, this protein provides an experimental basis for the development of a new type of oral therapy for type 2 diabetes.

## 2. Results

### 2.1. Analysis of Recombinant Transfer Vector pFastBac1-CTB-APSL

The *CTB-APSL* fragment was confirmed by sequencing and cloned into the pFastBac1 vector (data not shown).

### 2.2. Analysis of Recombinant Bacmid

**Figure 1:**
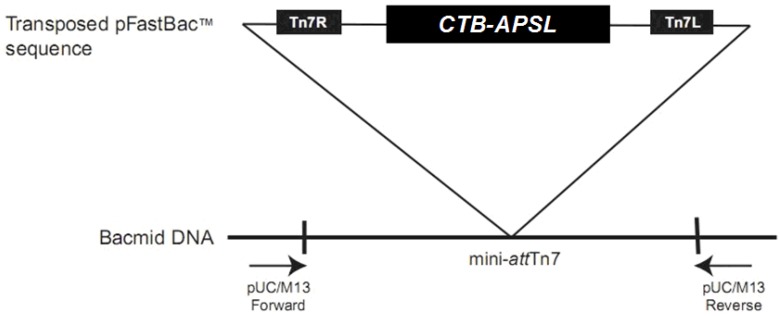
Transposition region analysis of pFastBac1-*CTB*-*APSL*. (CTB-APSL = cholera toxin B subunit and active peptide from shark liver).

The recombinant transfer vector was transformed into the *E. coli* DH10Bac-competent cells to generate the recombinant bacmid (Figure 1). The recombinant bacmid was then identified by sequencing using the M13 F (5′-GTTTTCCCAGTCACGAC-3′) and the M13 R (5′-CAGGAAACAGCTATGAC-3′) primers. 

### 2.3. Analysis of Recombinant Virus

**Figure 2:**
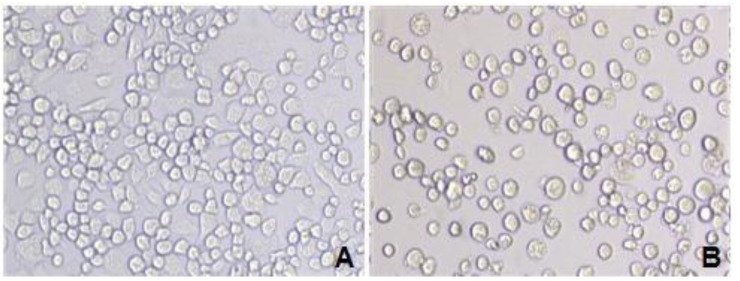
BmN cells transfected by recombinant virus under an optical microscope. **A**: Normal BmN cells (20 × 10); **B**: BmN cells transfected with recombinant virus (20 × 10).

Following transfection of BmN cells with recombinant virus, the cells became larger and rounded, and a number of them were in a suspended state (Figure 2). The recombinant virus was generated in the transfected BmN cells after 3–5 days, and the morphological changes of the transfected BmN cells were verified under an optical inverted microscope. 

**Figure 3:**
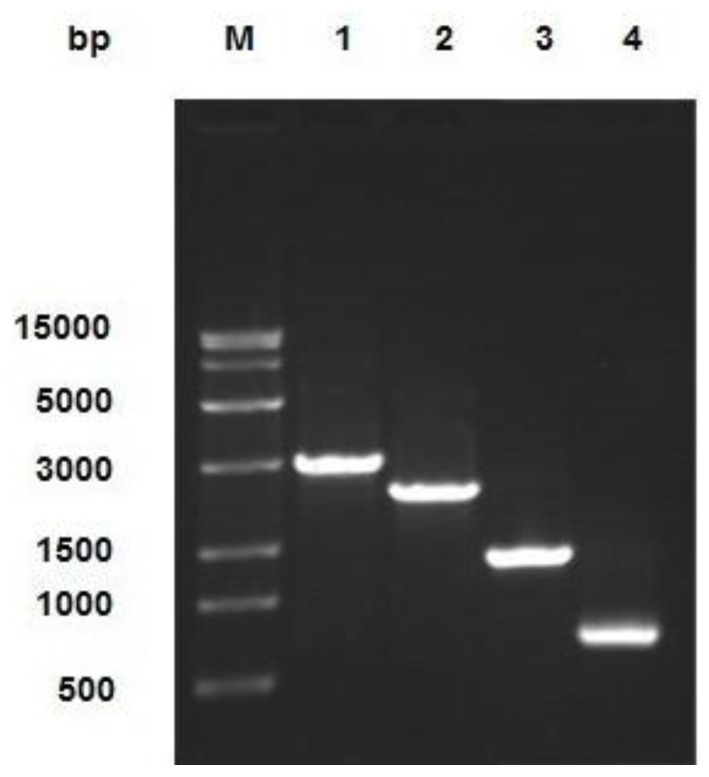
Identification of combinant virus DNA by PCR. The product was electrophoresed on 1% agarose gel. Lane M: Trans 15K DNA Marker; Lane 1: M13 F/M13 R PCR product; Lane 2: M13 F/P4 PCR product; Lane 3: P1/M13 R PCR product; Lane 4: P1/P4 PCR product.

Viral genomic DNA was extracted using a viral DNA purification kit, and the DNA was identified by PCR with the following primer pairs: M13 F/M13 R; M13 F/P4; P1/ M13 R; and P1/P4. The results of the PCR analysis, shown in Figure 3, are consistent with the expected results.

### 2.4. Expression of CTB-APSL Fusion Protein in Silkworm

**Figure 4:**
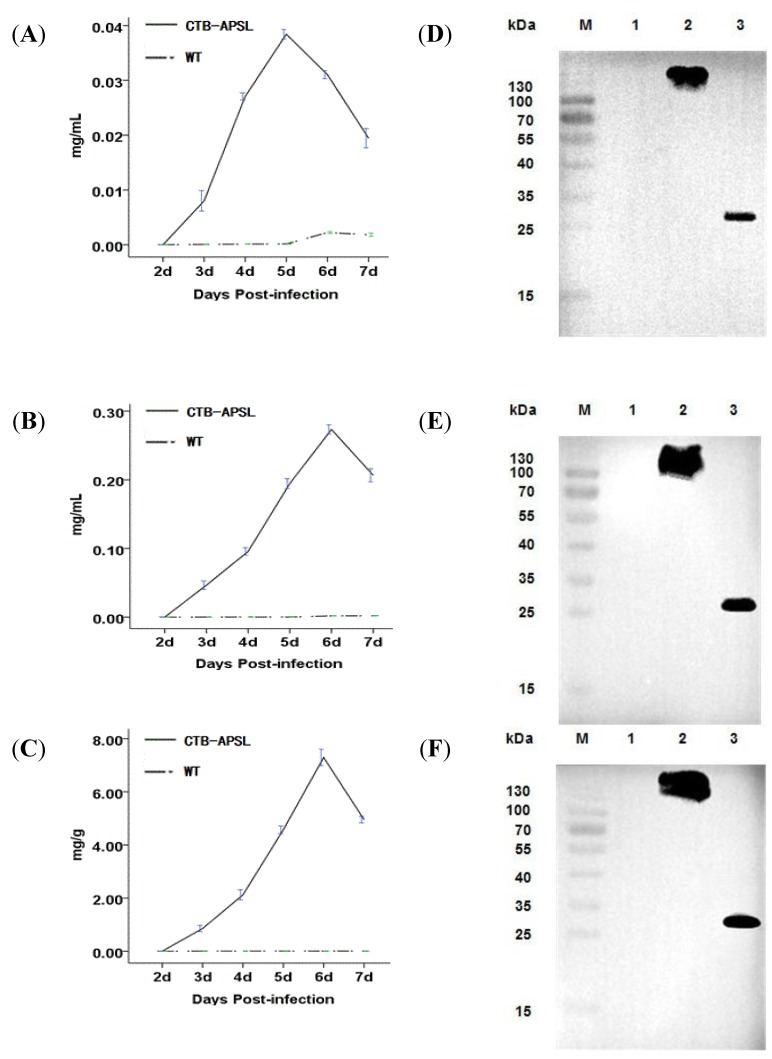
Quantitative analysis and western blotting analysis of CTB-APSL fusion protein. Analysis of fusion protein production levels in BmN cells (**A**), silkworm larvae hemolymph (**B**) and silkworm pupae freeze-dried powders (**C**). (**D**): Western blotting analysis of fusion protein expressed in infected BmN cells. Lane M: Pre-stained marker; Lane 1: Cell lysis supernatant infected by wild-type virus; Lane 2: Unboiled treated cell lysis supernatant infected by recombinant virus; Lane 3: Boiled treated cell lysis supernatant infected by recombinant virus. E: Western blotting analysis of fusion protein expressed in infected silkworm larvae emolymph. Lane M: Pre-stained marker; Lane 1: silkworm larvae hemolymph infected by wild-type virus; Lane 2: Unboiled treated silkworm larvae hemolymph infected by recombinant virus; Lane 3: Boiled treated silkworm larvae hemolymph infected by recombinant virus. F: Western blotting analysis of fusion protein expressed in infected silkworm pupae freeze-dried powders. Lane M: Pre-stained marker; Lane 1: silkworm pupae freeze-dried powders infected by wild-type virus; Lane 2: Unboiled treated silkworm pupae freeze-dried powders infected by recombinant virus; Lane 3: Boiled treated silkworm pupae freeze-dried powders infected by recombinant virus.

According to the Reed & Muench formula, the dilution of the recombinant virus was 1.44 × 10^9 ^(data not shown). After being infected with the recombinant virus, BmN cells and silkworm larvae and pupae displayed a series of viral infection symptoms and started to produce the CTB-APSL fusion protein. The ELISA results showed that the highest detectable level of the CTB-APSL fusion protein in cells yielded up to 0.039 mg/L ×10^6^ cells at the fifth day post-infection (Figure 4A). Moreover, at the sixth day post-infection, the maximum amount of CTB-APSL fusion protein reached 0.28 mg/mL in silkworm larvae hemolymph (Figure 4B) and 7.55 mg/g in pupae freeze-dried powders (Figure 4C). On average, 10 g pupae can yield 1 g of pupae freeze-dried powders when crushed and centrifuged at 12,000× *g* for 30 min. To detect the presence of ligand-antigen pentamers and monomers in cells, hemolymph, and freeze-dried powders, the presence of the CTB-APSL fusion protein was also examined in both unboiled and boiled samples. Fusion protein pentamers (approximately 130 kDa) were detected by Western blotting analysis of an unboiled sample incubated with CTB as the primary antibody. We found that the oligomeric fusion protein was dissociated into monomers by boiling for 10 min and subsequently migrated as a specific band with a molecular weight between 25–35 kDa. No immunospecific signal corresponding in molecular mass to the CTB-APSL fusion protein was detected in wild-type virus-infected silkworm samples (Figure 4D–F).

### 2.5. Affinity of CTB-APSL Fusion Protein for GM1 Ganglioside

**Figure 5:**
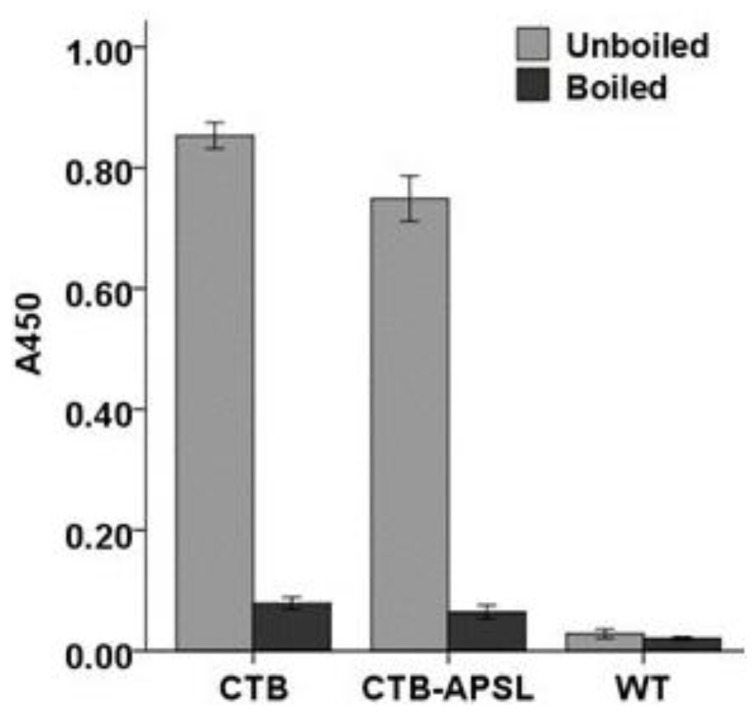
GM1 binding analysis of CTB-APSL fusion protein.

For confirmation of the specific affinity of the pentameric protein for GM1-ganglioside, we used a GM1-ELISA method with GM1-ganglioside as the capture molecule and the bacterial pentameric CTB to produce a standard curve. An increase in the concentration-specific absorption signal was observed, indicating that the fusion protein existed as a pentamer because only pentameric CTB can bind to GM1-ganglioside. However, the heat-treated fusion protein completely lost its affinity for GM1-ganglioside (Figure 5). The silkworm-derived CTB-APSL fusion protein exhibited the bioactivities and antigenic properties necessary for the purposes of this study. 

### 2.6. Changes in Body Weight, Fasting Blood Glucose, Kidney Coefficient and Spleen Coefficient

As shown in Table 1, the mice in the CTB-APSL group had increasing body weight after oral administration of CTB-APSL fusion protein for five weeks (*P* < 0.01). There was no significant difference in body weight among the wild-type group, the metformin group, and the diabetic group. It was observed that the CTB-APSL fusion protein had an effect of preventing weight loss in type 2 diabetic mice. The fasting blood glucose (FBG) levels were significantly decreased to 14.03 ± 2.09 mmol/L (*P* < 0.001) in the CTB-APSL group and 13.49 ± 1.88 mmol/L (*P* < 0.001) in the metformin group. Incidentally, the mice in the wild-type group also had a slight decrease in the level of FBG. It was demonstrated that CTB-APSL fusion protein was able to significantly reduce the FBG level in type 2 diabetic mice. The glycosylated hemoglobin (GHb) levels were significantly decreased in the CTB-APSL group (*P* < 0.001) and in the metformin group (*P* < 0.001). The GHb level in the wild-type group also had a slight decrease. It was demonstrated that CTB-APSL fusion protein was able to significantly reduce the GHb level in type 2 diabetic mice. The kidney coefficients were significantly decreased in the CTB-APSL group and the metformin group, and there were no significant differences in kidney coefficients between the wild-type group and the diabetic group. Spleen coefficients did not significantly differ among the experimental groups (data not shown).

**Table 1:** Metabolic and physiological parameters in experimental animals.

	Group Weight (g)	FBG (mmol/L)	GHb	Kidney Coefficient
Control	38.68 ± 1.40 ***	5.27 ± 0.58 ***	15.60 ± 2.27 ***	0.0172 ± 0.0009 ***
Diabetic	33.53 ± 1.84	18.14 ± 2.12	39.04 ± 5.67	0.0204 ± 0.0028
WT	34.33 ± 2.22	16.30 ± 1.97 *	34.13 ± 3.70 *	0.0192 ± 0.0017
CTB-APSL	36.38 ± 2.05 **	14.03 ± 2.09 ***	24.15 ± 4.22 ***	0.0182 ± 0.0013 **
Metformin	34.72 ± 1.85	13.49 ± 1.88 ***	23.53 ± 3.45 ***	0.0183 ± 0.0013 **

Data are means ± SEM for *n* = 12 per group. * *P* < 0.05 *vs.* diabetic group, ** *P* < 0.01 *vs.* diabetic group, *** *P* < 0.001 *vs.* diabetic group. FBG = fasting blood glucose: GHb = glycosylated hemoglobin.

### 2.7. FINS, HOMA-IR, HOMA-β, TG, TC, LDL, HDL, TNF-α and IL-6 Levels in Experimental Animals

**Figure 6:**
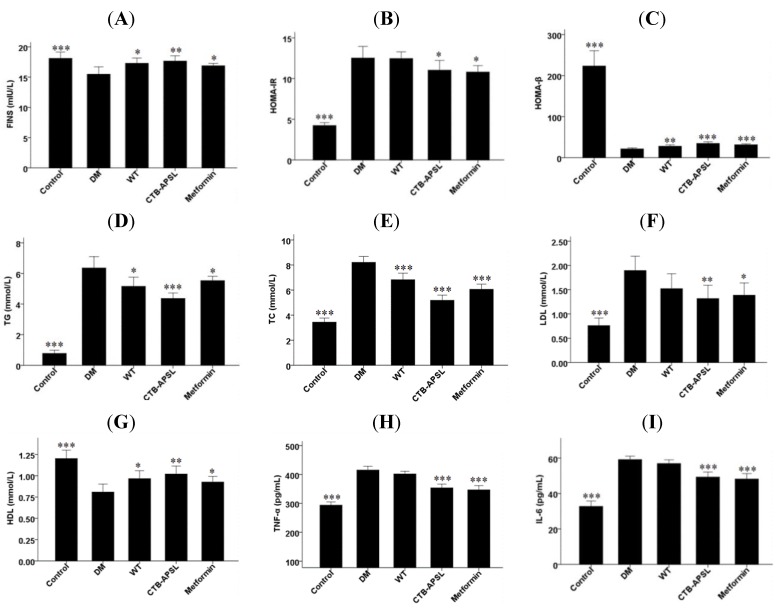
Fasting insulin (FINS), homeostasis model assessment of insulin resistance (HOMA-IR), homeostasis model assessment of β-cell function (HOMA-β), triglycerides (TG), total cholesterol (TC), low density lipoprotein (LDL), high density lipoprotein (HDL), tumor necrosis factor-α (TNF-α) and interleukin-6 (IL-6) levels in experimental animals. (**A**) FINS levels; (**B**) HOMA-IR levels; (**C**) HOMA-β levels; (**D**) TG levels; (**E**) TC levels; (**F**) LDL levels; (**G**) HDL levels; (**H**) TNF-α levels; (**I**) IL-6 levels.

The fasting insulin (FINS) level was significantly increased in the CTB-APSL group (*P* < 0.01) at the end of the fifth week, and the wild-type group and the metformin group also exhibited slight increases (Figure 6A). Thus, FINS was included in the formula (homeostasis model assessment of insulin resistance (HOMA-IR) = FINS × FBG/22.5; homeostasis model assessment of β-cell function (HOMA-β) = 20 × FINS/(FBG − 3.5)) to obtain the HOMA-IR and HOMA-β. HOMA-IR levels tended to be lower in the CTB-APSL group (*P* < 0.05) and the metformin group (*P* < 0.05) compared with the diabetic group. There was no significant difference in HOMA-IR levels between the wild-type group and the diabetic group (Figure 6B). HOMA-β levels in the CTB-APSL group (*P* < 0.001), the metformin group (*P* < 0.001) and the wild-type group (*P* < 0.01) all had a significant increase (Figure 6C). It was demonstrated that CTB-APSL fusion protein was able to effectively promote insulin secretion and to improve insulin resistance in type 2 diabetic mice. The triglycerides (TG) level was significantly decreased in the CTB-APSL group (*P* < 0.001) at the end of the fifth week, and the wild-type group and the metformin group also exhibited a slight reduction (Figure 6D). Total cholesterol (TC) levels in the CTB-APSL group (*P* < 0.001), the metformin group (*P* < 0.001) and the wild-type group(*P* < 0.001) were significantly increased (Figure 6E). The low density lipoprotein (LDL) level was significantly decreased in the CTB-APSL group (*P* < 0.01), and the metformin group also exhibited a slight reduction. There was no significant difference in LDL levels between the wild-type group and the diabetic group (Figure 6F). The high density lipoprotein (HDL) level was significantly increased in the CTB-APSL group (*P* < 0.01), and the wild-type group and the metformin group also exhibited a slight increase (Figure 6G). It was demonstrated that CTB-APSL fusion protein was able to significantly improve lipid metabolism in type 2 diabetic mice by reducing TG, TC and LDL levels and increasing the HDL level. The tumor necrosis factor-α (TNF-α) and interleukin-6 (IL-6) levels were both significantly decreased in the CTB-APSL group (*P* < 0.001) and in the metformin group (*P* < 0.001). There was no significant difference in TNF-α and IL-6 levels between the wild-type group and the diabetic group (Figure 6H,I). It was demonstrated that CTB-APSL fusion protein could effectively improve the inflammatory response in type 2 diabetic mice by reducing the levels of the inflammatory cytokines TNF-α and IL-6. 

### 2.8. Histological Analysis of Mouse Pancreatic, Hepatic and Nephritic Tissues in Experimental Animals

**Figure 7:**
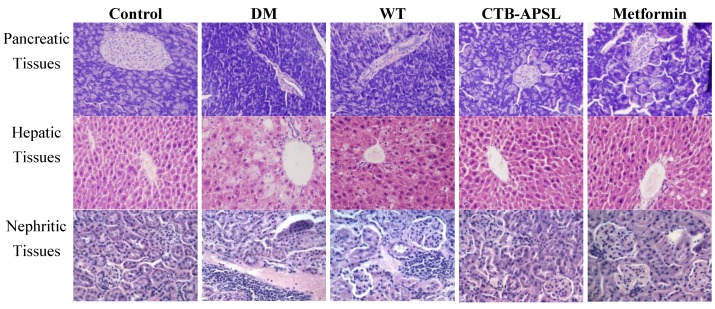
Histological analysis of mouse pancreatic tissues, hepatic tissues and nephritic tissues (200× HE).

Histological examination of mouse pancreatic tissues revealed islet atrophy, edge irregularities, and disordered pancreatic acinar cells in the diabetic group (Figure 7). After five weeks of treatment with CTB-APSL fusion protein, the pancreatic tissues from the CTB-APSL group exhibited regular edges and no distinct infiltration of the islet compared with the diabetic group. A similar improvement was observed in the metformin group. There were no significant pathological alterations in the pancreatic tissues from the wild-type group. Histological examination of mouse hepatic tissues revealed fatty degeneration, hepatocellular cloudiness and swelling and numbers of lipid droplets in hepatocytes in the diabetic group. After five weeks of treatment with CTB-APSL fusion protein, the pancreatic tissues from the CTB-APSL group exhibited liver cells arranged neatly and reduced or eradicated lipid droplets. A similar improvement was observed in the metformin group, although not to the same extent as in the CTB-APSL group. There was a slight improvement in fatty degeneration and hepatocellular cloudy swelling in the wild-type group. Histological examination of mouse nephritic tissues revealed inflammatory cells invasion, a few tubular epithelial cells deformation and necrosis, karyopyknosis and focal necrosis in the diabetic group. After five weeks of treatment with the CTB-APSL fusion protein, the pancreatic tissues from the CTB-APSL group exhibited renal corpuscle and renal tubular structures in good condition, no obvious inflammatory cell invasion and karyopyknosis compared with the diabetic group. A similar improvement was observed in the metformin group. There were no significant pathological alterations in the pancreatic tissues from the wild-type group. There were no significant pathological differences in the splenic tissues among the experimental groups (data not shown). 

## 3. Discussion

The clinical symptom of diabetes is hyperglycemia that is always accompanied by metabolism disorders of fat, protein and carbohydrates [3], which may easily produce serious complications affecting tissue lesions and some of the vital organs including the heart, blood vessels, nerves, eyes and kidneys [4]. Its acute complications are acute metabolic disorders including hyperosmolar nonketotic diabetic coma, ketoacidosis and lactic acid poisoning, while its main chronic complications are microvascular diseases (diabetic nephropathy, diabetic retinopathy) and macrovascular diseases (hypertension, heart disease, cerebrovascular accident and lower extremity vascular disease) and diabetic peripheral neuropathy [21,22]. It is predicted that the economic losses caused by diabetes and related complications will total up to $ 557.7 billion in China from 2005 to 2015 [23]. Because of its complex disease process, there is still no way to cure diabetes, and therefore, patients require lifelong treatment.

The major factors in the development of type 2 diabetes are islet β-cell functional defects and insulin resistance. At present, the major therapeutic drugs to treat type 2 diabetes are insulin secretagogues (sulfonylureas, meglitinides), insulin sensitizers (metformin, thiazolidinediones) and α-glucosidase inhibitors in both domestic and foreign markets. Meglitinides play a role by increasing insulin secretion but easily lead to hypoglycemia and weight gain, in addition to being expensive and having only short-term efficacy. Biguanide drugs are able to improve insulin sensitivity by inhibiting hepatic gluconeogenesis, enhancing glycolysis, and increasing the utilization of glucose in peripheral tissue, but are usually accompanied by side effects of gastrointestinal discomfort and risks of heart, liver and kidney failure [24]. Thiazolidinediones work by reducing glucose production, increasing the sensitivity of target tissues to insulin and improving insulin resistance, but the time to clinical effect can be protracted, and these medications may also cause peripheral edema and macular edema [25,26]. 

These drugs are used to treat type 2 diabetes by lowering blood glucose. Patients with type 2 diabetes need to take lifelong medicines, however, the effect of hypoglycemic medications has shown decreasing trends along with increasing the number of drugs. Above all, these drugs have failed to fundamentally repair dysfunctional and defected β cells. Islet β cell failure is also inevitable and will eventually lead to complete loss of islet β cell function. Therefore, the development of new medications to treat type 2 diabetes with high efficacy and low toxicity has great significance for the control of blood glucose, improving diabetes complications and improving the quality of life for patients. 

Although insulin therapy and hypoglycemic drugs can control blood glucose levels quickly and effectively and the mortality of acute complications due to diabetes also has been basically controlled, the chronic complications of treatment are inadequate. Chronic complications of diabetes should be the focus and core of the treatment of diabetes, yet unfortunately, traditional anti-diabetic drugs put emphasis on the changes in blood glucose and blood pressure while not effectively controlling the chronic complications of diabetes, resulting in a continued high mortality rate due to diabetes [27].

More and more studies suggest that endocrine-metabolic diseases have close relation with the liver. Betatrophin is a type of hormone primarily expressed in liver and fat tissues. Yi *et al.* found that betatrophin can regulate metabolism by increasing insulin production via an increase in β cell mass [28]. Jiang *et al.* reported that HSS was an anti-apoptotic factor during liver injury [29]. APSL was obtained from shark regenerated hepatic tissue, thus, it may also have the potential to treat endocrine-metabolic diseases, such as diabetes.

Studies found that prolonged exposure to high concentrations of free fatty acid (FFA) can cause lipid overload and increased apoptosis of β cells and reduced insulin secretion [30,31]. The CTB-APSL fusion protein could obviously improve lipid metabolism and thus has therapeutic effect on diabetes. A growing number of studies have confirmed that many types of inflammatory factors can predict the occurrence of type 2 diabetes, such as TNF-α and IL-6 [32,33]. In addition, inflammatory cytokines can cause insulin receptor signal transduction abnormalities, leading to the dysfunction of pancreatic β cells, participation in macrovascular and microvascular complications, and leading to retinopathy, non-alcoholic fatty liver and other serious consequences [34,35]. Meanwhile, clinical trials have confirmed that anti-inflammatory therapy can significantly improve type 2 diabetic patients with abnormal glucose metabolism [36].

*B. mori* has been used as a bioreactor for the production of recombinant proteins using the BmNPV expression system [37]. Baculoviruses do not infect vertebrate animals, and the system itself is safe [38]. These features make the silkworm system an ideal expression and delivery package for producing medicinal proteins for oral administration [39]. A major advantage of the BmNPV expression system is that it can be used to produce relatively large quantities of post-translationally modified heterologous proteins [40]. This expression system is inexpensive, convenient and produces large amounts of proteins. This system has been widely used to express recombinant proteins [41].

Our study demonstrates CTB-APSL fusion protein synthesis using the silkworm baculovirus expression vector system for the first time. Sharks are marine organisms, and products derived from sharks have a low toxicity when orally administered to mammals [42,43]. Silkworms contain a large number of natural protease inhibitors, and the oral formulation that we prepared contained silkworm-expressed CTB-APSL fusion protein, along with these protease inhibitors [44]. The CTB-APSL fusion protein was enclosed by lipidosome in the pupae, and these protease inhibitors and lipidosome may have a positive effect on helping the CTB-APSL fusion protein avoid the gastrointestinal digestive enzymes. In addition, the extracts from silkworm also have anti-diabetic effects [45].

In conclusion, our study demonstrates for the first time that CTB-APSL fusion protein has positive effects on the control of type 2 diabetes and effectively improves its complications, thus providing an experimental basis for the development of a new type of oral therapy for type 2 diabetes.

## 4. Experimental

### 4.1. Materials

DNA manipulation and PCR amplification kits were purchased from TaKaRa Biomedicals (Kyoto, Japan). The Viral Genomic DNA Purification Kit was purchased from Roche Co. (San Francisco, CA, USA). The pFasBac1 plasmid and the Lipofectamine 2000 Reagent were purchased from Invitrogen (Carlsbad, CA, USA). The *Bombyx mori* N cells (BmN cells), which originated from the ovary, were maintained in our laboratory and cultured at 27 °C in Sf-900 II Serum Free Medium Complete (GIBCO, Gran Island, NY, USA) containing 10% fetal bovine serum (GIBCO, Gran Island, NY, USA). The *E. coli* DH10Bac/BmNPV was constructed and supplied by our laboratory. The fifth instar silkworm larvae and diapausing pupae, Jingsong × Haoyue, were reared under a photoperiod schedule of 12 h light and 12 h darkness at 25 ± 1 °C and provided by Zhejiang Chinagene Biomedical Co., Ltd. (Haining, China). Male ICR mice were provided by Hangzhou Normal University Animal Center (Hangzhou, China) and were housed at the central animal facility, where they were screened for bacterial and viral pathogens. Streptozotocin (STZ), the rabbit anticholera toxin primary serum, bacterial CTB peptides, and monosialoganglioside-GM1 were purchased from Sigma Co. (St. Louis, MO, USA).

### 4.2. Construction of Recombinant Transfer Vector

Four synthetic oligonucleotides were designed to amplify the CTB-APSL gene. Using the pET-28a-APSL plasmid and the pGEX-4T-1-CTB plasmid as templates, the fusion genes based on the overlap region (GPGP) were amplified by splice overlap extension PCR (SOE-PCR). The CTB-APSL fusion gene was inserted into the transfer vector pFastBac1, and then the recombinant vector was verified by PCR identification and fragment sequencing. 

### 4.3. Transfection and Acquisition of the Recombinant Virus

To obtain the recombinant virus, the recombinant bacmid vector was transfected into BmN cells using the Lipofectamine 2000 Reagent (Invitrogen, Carlsbad, CA, USA), and the wild-type bacmid vector was used as a control. The recombinant virus was generated in the transfected BmN cells after 5 days, and the titer of the virus was calculated using the Reed-Muench method. The genome of recombinant virus was obtained with the Viral Genomic DNA Purification Kit (Axygen, Union City, CA, USA) and analyzed by PCR identification and fragment sequencing.

### 4.4. Expression and Collection of the CTB-APSL Fusion Protein

BmN cells (4 × 10^6^) were infected with recombinant virus at an MOI of 10 and collected at 2–7 days after infection. The harvested cells were resuspended in 0.2 mL of phosphate buffered saline (PBS) before being disrupted using gentle sonication for 5 min on ice. The samples were stored at −80 °C after centrifugation until further analysis. Fifth instar silkworm larvae and diapausing pupae were infected with recombinant viral solution (1 × 10^7^ pfu/mL) by subcutaneous injection. Hemolymph from larvae was collected at 2–7 days post-inoculation and centrifuged at 12,000× *g* for 30 min at 4 °C to remove the insoluble impurities. The hemolymph samples were then stored at −80 °C. The pupae were collected at 2–7 days post-inoculation and crushed. The pupal mash was centrifuged at 12,000× *g* for 30 min at 4 °C to remove most of the top lipid layer and the bottom layer of debris. The upper solution was centrifuged three times as described above to remove the remaining lipids and debris. The pupal supernatant was stored at −80 °C and was then freeze-dried to powder. Wild-type virus was used as a control.

### 4.5. Western Blotting and ELISA Assay

To detect the expression of monomeric or pentameric fusion proteins, the cell lysis supernatant samples, hemolymph samples and pupal supernatant samples were subjected to 12% SDS-PAGE. Samples were either boiled or loaded directly on the gel. The separated protein bands were transferred to an NC ﬁlter membrane. The membrane was blocked for 2 h in Tris-buffered saline (TBS) containing 5% nonfat dry milk and incubated with rabbit anticholera toxin anti-serum (Sigma, St. Louis, MO, USA). After it was washed in Tris-buffered saline with Tween (TBST, 0.5% Tween-20), the corresponding secondary antibodies were incubated for 2 h. Detection of the immunoreaction was performed with an enhanced chemiluminescence (ECL) Western blotting kit (Advansta, Menlo Park, CA, USA).

The CTB-APSL fusion protein levels in BmN cells, silkworm larvae and pupae were determined by semiquantitative ELISA assay. A 96-well microtiter plate (JET, Canada) was loaded with serial dilutions of the cell-lysed supernatant, hemolymph and pupal supernatant in bicarbonate buffer, pH 9.6 (15 mmol/L Na_2_CO_3_, 35 mmol/L NaHCO_3_), and incubated overnight at 4 °C. The plate was washed three times in PBS containing 0.05% Tween-20 (PBST). The plate was incubated in a 1:8000 dilution of rabbit anti-cholera toxin primary antibody (100 µL/well) at 37 °C for 2 h, followed by three washes with PBST. The plate was then incubated with a 1:2000 dilution of anti-rabbit IgG conjugated with horseradish peroxidase (Biosharp, Seoul, Korea) (100 µL/well) for 2 h at 37 °C and washed three times with PBST. Finally, the chromogenic substrate TMB (Innoreagents, China) (100 µL/well) was added to the wells, and the plate was incubated for 20 min at 37 °C to develop color, followed by the addition of 2 M H_2_SO_4_ (50 µL/well) to stop the reaction. The absorbance at 450 nm was measured in a Multiskan MS ELISA plate reader (Labsystems, Finland). 

### 4.6. GM1 Binding Assay

A GM1-ELISA was performed to detect the affinity of silkworm-derived CTB-APSL fusion protein for GM1-ganglioaide. The microtiter plate was coated with monosialoganglioside-GM1 (Sigma, St. Louis, MO, USA) by incubating the plate with 50 µL/well of GM1 (10 µg/mL) in methanol at 4 °C overnight. The same dilutions of either wild-type baculovirus-infected sample or bacterial CTB were used as negative and positive controls, respectively. The remainder of the procedure was identical to the ELISA assay described above. 

### 4.7. Diabetic Mouse Models

All animal protocols and procedures were approved by the IACUC (Institutional Animal Care and Use Committee) at Hangzhou Normal University. One hundred ICR male mice, weighing 18–22 g, were housed in a room with a 12:12-h artificial light cycle, a temperature of 20 °C ± 2 °C, and a humidity of 55% ± 5%. The animals had free access to diet and tap water throughout the experiment. Twelve mice were selected randomly as the control group and fed a standard diet. The rest of the mice were fed a high-fat and high-sugar diet for four weeks. After four weeks, all of the mice except for the control group were injected with STZ (80 mg/kg body wt) intraperitoneally 12 h after fasting. The levels of FBG were measured by a Roche glucometer (Accu-Chek Advantage, Mannheim, Germany) 72 h after STZ administration. Only the mice with FBG levels over 11.1 mmol/L were selected for the experiment.

### 4.8. Drug Delivery

The type 2 diabetic mice were divided into the following groups: the diabetic group (*n* = 12), the wild-type group (*n* = 12), the CTB-APSL group (*n* = 12), and the metformin group (*n* = 12). The mice in the control and the diabetic groups were treated with normal saline (i.g. 0.2 mL/10 g·day wt) only, the mice in the CTB-APSL group were treated with CTB-APSL (i.g. 100 mg /kg·day wt), the mice in the wild-type group were treated with freeze-dried powder from pupae infected with wild-type virus, and the mice in the metformin group were treated with metformin (i.g. 200 mg/kg·day wt).

### 4.9. Specimen Collection and Biochemical Indicator

Body weight and the FBG levels were measured weekly on the 10th, 17th, 24th, 31st, and 38th day after STZ administration. The FBG levels were measured via tail vein blood using a glucometer. At the end of the fifth week, all of the mice were executed and blood was collected, then centrifuged at 3000× *g* for 20 min at 4 °C to separate serum. The serum was then stored at −80 °C after repackaging. Pancreases, livers, kidneys and spleens of all the experimental animals were quickly removed, after which the kidneys and spleens were weighed, and then all specimens were fixed in 4% paraformaldehyde. GHb, FINS, TG, TC, LDL-C, HDL-C, TNF-α and IL-6 in serum were measured with commercial enzyme-linked immunosorbent assay kits purchased from Jinan Linuo Biological Co. (Jinan, China) (FINS, TNF-α and IL-6 kits) and Nanjing Jiancheng Technological Co. (Nanjing, China) (GHb, TG, TC, HDL-C and LDL-C kits).

### 4.10. Histological Analysis

The specimens were fixed in 4% paraformaldehyde for two weeks, conventionally washed, and then processed for conventional paraffin embedding. Sections (8 μm) were mounted on glass slides, dewaxed in xylene, rehydrated through graded alcohols, washed in distilled water and stained with hematoxylin and eosin (HE). All slides were examined under a microscope.

### 4.11. Statistical Analysis

All results are expressed as the mean ± SEM. Statistical analysis of the data for multiple comparisons was performed by analysis of variance (ANOVA). For single comparisons, the significance of differences between means was determined by *t*-tests. Values of *P* < 0.05 were considered statistically significant, and a value of *P* < 0.001 was considered statistically most significant.

## 5. Conclusions

In this study, CTB-APSL fusion gene was cloned and expressed using the Bac-to-Bac baculovirus expression system. The fusion protein which expressed in the silkworm baculovirus expression vector system existed as a pentamer, thus had bioactivities and could bind to GM1-ganglioside. Studies have shown that CTB-APSL has a good effect on anti-type 2 diabetes in mice, and effectively improves complications. The results of this study provide an experimental basis for the development of new type 2 diabetic oral drugs.
